# Painful sexual intercourse, quality of life and sexual function in patients with endometriosis: not just deep dyspareunia

**DOI:** 10.1007/s00404-024-07643-7

**Published:** 2024-07-25

**Authors:** Simona Del Forno, Arianna Raspollini, Marisol Doglioli, Anna Andreotti, Emanuela Spagnolo, Jacopo Lenzi, Giulia Borghese, Diego Raimondo, Alessandro Arena, Elena Rodriguez, Alicia Hernandez, Francesca Govoni, Renato Seracchioli

**Affiliations:** 1 Division of Gynaecology and Human Reproduction Physiopathology, IRCCS Azienda Ospedaliero-Universitaria di Bologna, Via Massarenti 13, Bologna, Italy; 2 Department of Medical and Surgical Sciences (DIMEC), University of Bologna, Bologna, Italy; 3 Department of Obstetrics and Gynecology, Hospital Universitario La Paz, Madrid, Spain; 4 Department of Biomedical and Neuromotor Sciences, University of Bologna, Bologna, Italy

**Keywords:** Quality of life, Sexual function, Pain, Endometriosis

## Abstract

**Purpose:**

To evaluate the prevalence of deep and superficial dyspareunia in women with diagnosis of endometriosis. Secondly, to assess the temporal relation between deep and superficial dyspareunia in women reporting both symptoms (concomitant dyspareunia) and the impact on quality of life (QoL) and sexual function.

**Methods:**

This is a cross-sectional cohort study that included fertile women with diagnosis of endometriosis. Enrolled subjects reported pain symptoms including dyspareunia and its temporal onset and completed two one-time validated questionnaires regarding sexual function (Female Sexual Function Index) and QoL (International QoL Assessment SF-36).

**Results:**

Among the 334 enrolled patients, 75.7% (95%) reported dyspareunia. Women were divided into four groups according to the presence and type of dyspareunia: isolated superficial dyspareunia (6.3%), isolated deep dyspareunia (26.0%), concomitant dyspareunia (43.4%) and no dyspareunia (24.3%). Women with concomitant dyspareunia reported higher NRS scores than women with isolated dyspareunia or no dyspareunia (P ≤ 0.001). The majority of women with concomitant dyspareunia (56.6%) reported that deep dyspareunia developed before superficial dyspareunia. Women with concomitant dyspareunia reported worse QoL and worse sexual function than women with isolated dyspareunia or without dyspareunia (P ≤ 0.001).

**Conclusion:**

Dyspareunia is a common symptom in women with endometriosis, with many reporting concomitant deep and superficial dyspareunia. Concomitant dyspareunia can significantly impact sexual function and quality of life (QoL). Therefore, it is crucial to investigate dyspareunia thoroughly and differentiate between its types to tailor effective therapeutic strategies.

**Supplementary Information:**

The online version contains supplementary material available at 10.1007/s00404-024-07643-7.

## What does this study add to the clinical work

**Table:** 

Endometriosis is a chronic inflammatory disease responsible of pain symptoms, such as dyspareunia. This study found that many women affected by endometriosis report concomitant deep and superficial dyspareunia. Concomitant dyspareunia can significantly impact sexual function and quality of life (QoL)	

## Introduction

Endometriosis is a chronic inflammatory disease affecting 10–15% of fertile age women, characterized by the presence of endometrial tissue outside the uterine cavity [1]. The disease may cause severe pain symptoms, such as dysmenorrhea, chronic pelvic pain, dysuria, dyschezia and dyspareunia [1]. Endometriosis may have a negative impact on quality of life (QoL) due to pain symptoms that can compromise daily activities, work productivity and social relationships [2–6]. Moreover, women with endometriosis may experience sexual dysfunction, mainly due to painful sexual intercourse (dyspareunia). Research indicates a reduction in both frequency and quality of sexual activity among these women, often accompanied by feelings of guilt and diminished femininity, thereby exacerbating challenges in forming intimate connections [7, 8]. The prevalence of dyspareunia in women with endometriosis is estimated to be around half of the affected women. [9]. It can be categorized into superficial (pain occurring in or around the vaginal entrance), and deep (pain in the vagina and pelvis during and/or after sexual intercourse) [10, 11]. While deep dyspareunia has garnered substantial attention in literature, attributed largely to mechanical pressure on endometriotic lesions or tissue rigidity, superficial dyspareunia and its association with endometriosis remains relatively understudied [10–13]. Delineating the distinction between deep and superficial dyspareunia may be helpful for clinicians in order to offer women different and combined treatments, such as pelvic floor physiotherapy other than medical and surgical therapies [12–14].

This study aims to elucidate the prevalence of deep and superficial dyspareunia among women with endometriosis across two referral centers, elucidating their respective impacts on QoL and sexual function. Furthermore, we aim to explore the temporal relationship between the onset of deep and superficial dyspareunia in patients experiencing both symptoms, providing insights crucial for comprehensive management strategies.

## Materials and methods

### Study protocol, selection criteria and study outcomes

This study was designed as a bicentric, prospective, cross sectional study following an *a priori* defined study protocol.

All consecutive patients who attended outpatient clinics of the Division of Gynecology and Human Reproduction Physiopathology, IRCCS Azienda Ospedaliero-Universitaria di Bologna, Italy, and the Department of Obstetrics and Gynecology, Hospital Universitario La Paz, Madrid and were diagnosed as having endometriosis from June to December 2022 were invited to participate to the study.

Inclusion criteria were: age between 18 and 50 years old, clinical and ultrasonographic diagnosis of endometriosis and a history of engaging in penetrative vaginal sexual relations with a penis, sexual objects, or fingers at least once in their life. Exclusion criteria were: menopausal status and no history of penetrative vaginal sexual relations with a penis, sexual objects, or fingers. Women were included regardless of their current sexual activity to ensure that those with severe dyspareunia, which may have led to cessation of sexual activity, were not excluded from the study.

Demographic and clinical data from medical history were collected by interview, and bimanual gynecologic examination and ultrasound evaluation were performed by expert operators and following the International Deep Endometriosis Analysis (IDEA) consensus [15]. After the gynecologic consultation, 2 clinical researchers (A.R. and S.D.F.) invited eligible patients to participate in the study and collected relative informed consent. Two validated paper questionnaires were administered to assess sexual function and QoL.

Primary study outcome was the prevalence of deep and superficial dyspareunia among women with endometriosis across two referral centers, and secondary study outcomes were the evaluation of the temporal relationship between the onset of deep and superficial dyspareunia and their impact on QoL and sexual function.

### Demographic and clinical factors

For all enrolled women, demographic data and a detailed clinical history were recorded before ultrasound scan in the Case Report Form (CRF). In particular, we recorded: age, body mass index, smoking, age at menarche, localization of endometriosis, current hormonal treatment, previous surgery, endometriosis associated symptoms (dysmenorrhea, dyschezia, dysuria, chronic pelvic pain, dyspareunia and ovulatory pain) and their severity assessed using the Numerical Rating Scale (NRS) [8]. Each symptom was classified as mild (NRS 1 − 5) or moderate-severe (NRS 6 − 10). Patients received detailed verbal explanation of the differences between deep and superficial dyspareunia. Dyspareunia was classified as isolated superficial dyspareunia or isolated deep dyspareunia if only one symptom was present (NRS ≥ 1). For women reporting both symptoms, dyspareunia was classified as concomitant dyspareunia, and they were asked to specify which symptom arose first. Participants were divided into four groups according to the reported symptom: (a) isolated superficial dyspareunia, (b) isolated deep dyspareunia, (c) concomitant dyspareunia, (d) no dyspareunia.

Additionally, participants were asked to specify the number of sexual intercourses per month (penetrative vaginal sexual relations with a penis, sexual objects, or fingers) and were classified as sexually active (≥ 1 sexual intercourse per month) or not sexually active (0 sexual intercourses per month).

Two validated paper questionnaires were administered to assess sexual function (Female Sexual Function Index, FSFI) and QoL (International Quality of Life Assessment, SF-36) [15–19]. The SF-36 includes 8 subscales evaluating physical functioning, physical role functioning, bodily pain, general health perceptions, vitality, social functioning, social role functioning, and mental health. Moreover, participants are asked to compare their self-rated health to the previous year to evaluate perceived changes in health (SF-36 question #2). The FSFI is a 19-item self-report measure assessing overall sexual function and its primary components: sexual desire, arousal, orgasm, pain, and satisfaction [18, 19].

### Statistical analysis

Categorical variables were reported as numbers and percentages. Discrete variables were presented as numbers and percentages or as medians with interquartile ranges. Continuous variables were expressed as means ± standard deviations. Differences among the four study groups (isolated superficial dyspareunia, isolated deep dyspareunia, both superficial and deep dyspareunia, and no dyspareunia) in demographic and clinical characteristics were assessed using the Chi-squared test, Fisher’s exact test, or Kruskal–Wallis test, depending on the variable type.

The association between the intensity of dyspareunia (measured by NRS) and SF-36 scores was assessed using Spearman’s rank correlation coefficient (ρ). All statistical analyses were performed using Stata 17 (StataCorp, 2021. Stata Statistical Software: Release 17. College Station, TX: StataCorp LLC). The significance level was set at 0.05, and all tests were two-tailed.

For sample size determination, we assumed a prevalence of 50% for both deep and superficial dyspareunia, which maximizes variance (0.5 × 0.5 = 0.25) and thus the number of patients required for analysis [20]. Assuming that 30% of women with endometriosis experience both types of dyspareunia, the overall prevalence of either type was estimated by adding the individual probabilities and subtracting the probability of both conditions co-occurring, resulting in 50% + 50%–30% = 70%. Using this prevalence, we calculated that enrolling at least 323 patients would provide a precision of ± 5% for a 95% two-sided normal-based confidence interval (95% CI: 71.2–80.3) [20, 21].

### Ethical statement

The study received approval from the Institutional Review Board (CE-AVEC 254/2022/Oss/AOUBo) and was carried out in accordance with the Helsinki Declaration. All patients signed an informed consent for the use of their anonymized data for the study.

## Results

**Fig. 1:**
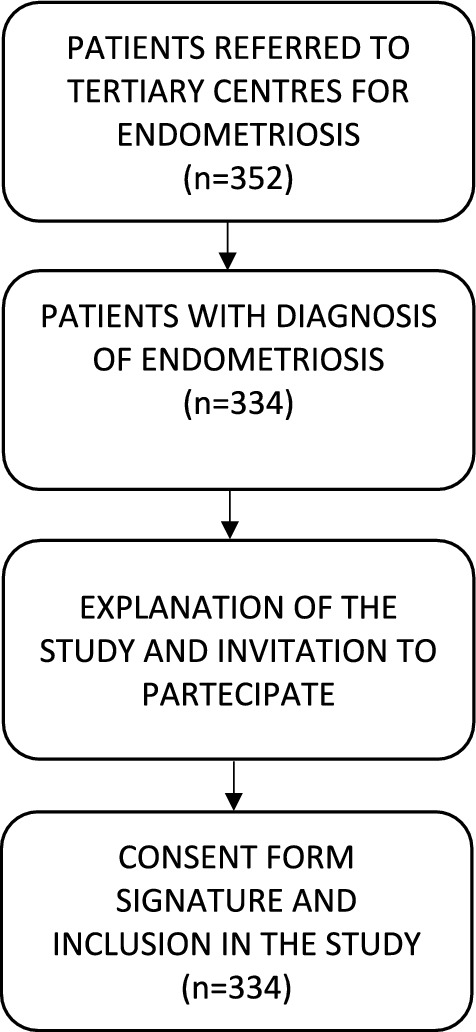
Flowchart: patient’s selection

The study enrolled 334 consecutive patients: 238 at Bologna’s center and 96 at Madrid’s center (Fig. 1). Dyspareunia was reported by 75.7% of women, 21 (6.3%) reported isolated superficial dyspareunia, 87 (26.0%) reported isolated deep dyspareunia, and the majority (145/334, 43.4%) reported concomitant dyspareunia.

Demographic, clinical and ultrasound characteristics of the study population, overall and by study groups (isolated superficial dyspareunia, isolated deep dyspareunia, concomitant deep and superficial dyspareunia, no dyspareunia) are showed in Table 1.

**Table 1:** Demographic and clinical characteristics of the study sample, overall and by study group

Characteristic	All	Isolated superficial dyspareunia	Isolated deep dyspareunia	Concomitant deep and superficial dyspareunia	No dyspareunia	*P*-value
	(*n* = 334)	(*n* = 21)	(*n* = 87)	(*n* = 145)	(*n* = 81)	
Age group, y						0.17
≤ 35	93 (27.8%)	5 (23.8%)	27 (31.0%)	49 (33.8%)	12 (14.8%)	
36 to 40	88 (26.3%)	7 (33.3%)	23 (26.4%)	31 (21.4%)	27 (33.3%)	
41 to 45	76 (22.8%)	6 (28.6%)	19 (21.8%)	32 (22.1%)	19 (23.5%)	
> 45	77 (23.1%)	3 (14.3%)	18 (20.7%)	33 (22.8%)	23 (28.4%)	
BMI class, kg/m^2^						0.36
< 25	247 (74.0%)	13 (61.9%)	70 (80.5%)	106 (73.1%)	58 (71.6%)	
25 to < 30	58 (17.4%)	5 (23.8%)	13 (14.9%)	23 (15.9%)	17 (21.0%)	
≥ 30	29 (8.7%)	3 (14.3%)	4 (4.6%)	16 (11.0%)	6 (7.4%)	
Type of endometriosis						
Adenomyosis	180 (53.9%)	10 (47.6%)	48 (55.2%)	83 (57.2%)	39 (48.1%)	0.55
Posterior DIE	134 (40.1%)	6 (28.6%)	33 (37.9%)	62 (42.8%)	33 (40.7%)	0.62
Ovarian	106 (31.7%)	6 (28.6%)	24 (27.6%)	51 (35.2%)	25 (30.9%)	0.66
Anterior DIE	6 (1.8%)	0 (0.0%)	2 (2.3%)	2 (1.4%)	2 (2.5%)	0.84
Other	10 (3.0%)	2 (9.5%)	1 (1.1%)	5 (3.4%)	2 (2.5%)	0.24
DIE site						
Rectum	93 (27.8%)	6 (28.6%)	23 (26.4%)	40 (27.6%)	24 (29.6%)	0.97
Uterosacral ligaments	45 (13.5%)	1 (4.8%)	14 (16.1%)	19 (13.1%)	11 (13.6%)	0.65
Sigma	32 (9.6%)	3 (14.3%)	10 (11.5%)	16 (11.0%)	3 (3.7%)	0.14
Torus	15 (4.5%)	1 (4.8%)	3 (3.4%)	9 (6.2%)	2 (2.5%)	0.58
Rectovaginal septum	8 (2.4%)	0 (0.0%)	1 (1.1%)	5 (3.4%)	2 (2.5%)	0.76
Vagina	6 (1.8%)	0 (0.0%)	1 (1.1%)	3 (2.1%)	2 (2.5%)	0.92
Bladder	5 (1.5%)	0 (0.0%)	2 (2.3%)	2 (1.4%)	1 (1.2%)	0.90
Ureters	2 (0.6%)	1 (4.8%)	1 (1.1%)	0 (0.0%)	0 (0.0%)	0.07
Parametrium	6 (1.8%)	0 (0.0%)	0 (0.0%)	2 (1.4%)	4 (4.9%)	0.11
HT						0.27
No	114 (34.1%)	8 (38.1%)	33 (37.9%)	41 (28.3%)	32 (39.5%)	
Yes	220 (65.9%)	13 (61.9%)	54 (62.1%)	104 (71.7%)	49 (60.5%)	
Type of HT						0.30
No HT	114 (34.1%)	8 (38.1%)	33 (37.9%)	41 (28.3%)	32 (39.5%)	
E/P	61 (18.3%)	5 (23.8%)	15 (17.2%)	30 (20.7%)	11 (13.6%)	
P	122 (36.5%)	7 (33.3%)	27 (31.0%)	62 (42.8%)	26 (32.1%)	
LNG-IUD	37 (11.1%)	1 (4.8%)	12 (13.8%)	12 (8.3%)	12 (14.8%)	
Previous surgery						0.76
No	138 (41.3%)	8 (38.1%)	36 (41.4%)	64 (44.1%)	30 (37.0%)	
Yes	196 (58.7%)	13 (61.9%)	51 (58.6%)	81 (55.9%)	51 (63.0%)	
Type of surgery						0.61
No surgery	138 (41.3%)	8 (38.1%)	36 (41.4%)	64 (44.1%)	30 (37.0%)	
Ovarian	74 (22.2%)	5 (23.8%)	14 (16.1%)	35 (24.1%)	20 (24.7%)	
DIE	45 (13.5%)	3 (14.3%)	17 (19.5%)	14 (9.7%)	11 (13.6%)	
Ovarian and DIE	76 (22.8%)	5 (23.8%)	20 (23.0%)	32 (22.1%)	19 (23.5%)	
Superficial	1 (0.3%)	0 (0.0%)	0 (0.0%)	0 (0.0%)	1 (1.2%)	
Previous pregnancies						0.11
No	280 (83.8%)	18 (85.7%)	70 (80.5%)	129 (89.0%)	63 (77.8%)	
Yes	54 (16.2%)	3 (14.3%)	17 (19.5%)	16 (11.0%)	18 (22.2%)	
Smoker						0.60
No	243 (72.8%)	17 (81.0%)	65 (74.7%)	106 (73.1%)	55 (67.9%)	
Yes	91 (27.2%)	4 (19.0%)	22 (25.3%)	39 (26.9%)	26 (32.1%)	
Sexual intercourses/month						0.09
0	106 (31.7%)	7 (33.3%)	18 (20.7%)	53 (36.6%)	28 (34.6%)	
1	84 (25.1%)	8 (38.1%)	24 (27.6%)	35 (24.1%)	17 (21.0%)	
2	63 (18.9%)	3 (14.3%)	19 (21.8%)	26 (17.9%)	15 (18.5%)	
3	34 (10.2%)	2 (9.5%)	12 (13.8%)	11 (7.6%)	9 (11.1%)	
4	16 (4.8%)	1 (4.8%)	4 (4.6%)	4 (2.8%)	7 (8.6%)	
≥ 5	31 (9.3%)	0 (0.0%)	10 (11.5%)	16 (11.0%)	5 (6.2%)	
Dysmenorrhea, NRS						0.002*
0	172 (51.5%)	13 (61.9%)	35 (40.2%)	73 (50.3%)	51 (63.0%)	
1 to 5	58 (17.4%)	2 (9.5%)	20 (23.0%)	18 (12.4%)	18 (22.2%)	
6 to 10	104 (31.1%)	6 (28.6%)	32 (36.8%)	54 (37.2%)	12 (14.8%)	
Dyschezia, NRS						0.002*
0	245 (73.4%)	18 (85.7%)	62 (71.3%)	95 (65.5%)	70 (86.4%)	
1 to 5	41 (12.3%)	0 (0.0%)	16 (18.4%)	19 (13.1%)	6 (7.4%)	
6 to 10	48 (14.4%)	3 (14.3%)	9 (10.3%)	31 (21.4%)	5 (6.2%)	
Dysuria, NRS						0.001*
0	292 (87.4%)	20 (95.2%)	77 (88.5%)	117 (80.7%)	78 (96.3%)	
1 to 5	23 (6.9%)	0 (0.0%)	6 (6.9%)	17 (11.7%)	0 (0.0%)	
6 to 10	19 (5.7%)	1 (4.8%)	4 (4.6%)	11 (7.6%)	3 (3.7%)	
Chronic pelvic pain, NRS						< 0.001*
0	196 (58.7%)	16 (76.2%)	49 (56.3%)	65 (44.8%)	66 (81.5%)	
1 to 5	66 (19.8%)	4 (19.0%)	22 (25.3%)	32 (22.1%)	8 (9.9%)	
6 to 10	72 (21.6%)	1 (4.8%)	16 (18.4%)	48 (33.1%)	7 (8.6%)	
Ovulation pain, NRS						0.12
0	217 (65.0%)	15 (71.4%)	53 (60.9%)	87 (60.0%)	62 (76.5%)	
1 to 5	56 (16.8%)	3 (14.3%)	17 (19.5%)	24 (16.6%)	12 (14.8%)	
6 to 10	61 (18.3%)	3 (14.3%)	17 (19.5%)	34 (23.4%)	7 (8.6%)	

*P-value ≤ 0.05

^a^*BMI* body mass index, *HT* hormone therapy, *E/P* combined estrogen-progestin therapy, *P* progestin therapy, *LNG-IUD* levonorgestrel-releasing intrauterine device, *DIE* deep infiltrating endometriosis, *NRS* numerical rating scale

The group of patients with concomitant dyspareunia showed a higher frequency of moderate-severe dysmenorrhea (P = 0.002), dyschezia (P = 0.002), dysuria (P = 0.001), chronic pelvic pain (P =  < 0.001) (Table 1). Dyspareunia severity is reported in Supplementary Table 1. Women with concomitant dyspareunia reported higher NRS scores (in 83.4% of patients the symptom was moderate-severe) than women with isolated dyspareunia or no dyspareunia (P =  < 0.001) (Supplementary Table 1).

### Temporal relationship of dyspareunia symptoms

Among women reporting concomitant dyspareunia, 82 out of 145 (56.6%) indicated that deep dyspareunia occurred first, while 63 (43.4%) reported that superficial dyspareunia occurred first.

### Quality of life (QoL) assessment

Scores from the SF-36 (physical functioning, physical role functioning, bodily pain, general health, vitality, social functioning, social role functioning, mental health) are reported in Table 2. Physical functioning, physical role functioning, bodily pain, general health and social role functioning were significantly impaired in women with concomitant dyspareunia (P =  < 0.001), while vitality and social functioning were reduced in women with concomitant dyspareunia and isolated deep dyspareunia (P =  < 0.001).

**Table 2:** Mean ± standard deviation of eight 36-Item Short Form Survey (SF-36) subscale scorings overall and by study group

Scale	All	Isolated superficial dyspareunia	Isolated deep dyspareunia	Concomitant deep and superficial dyspareunia	No dyspareunia	*P*-value
	(*n* = 334)	(*n* = 21)	(*n* = 87)	(*n* = 145)	(*n* = 81)	
Physical functioning	86.9 ± 20.4	89.3 ± 14.4	91.6 ± 13.8	80.4 ± 25.2	92.7 ± 14.3	< 0.001*
Physical role functioning	68.3 ± 38.2	77.4 ± 33.5	73.0 ± 35.2	57.2 ± 40.2	80.6 ± 33.5	< 0.001*
Bodily pain	63.7 ± 27.7	68.9 ± 26.1	67.8 ± 26.5	55.2 ± 27.3	73.1 ± 25.9	< 0.001*
General health	54.9 ± 21.2	49.0 ± 20.0	55.1 ± 20.3	48.9 ± 20.3	66.9 ± 19.3	< 0.001*
Vitality	49.0 ± 18.2	52.4 ± 16.6	49.4 ± 18.1	44.6 ± 17.6	55.3 ± 17.8	< 0.001*
Social functioning	61.1 ± 26.1	63.7 ± 30.3	63.4 ± 21.8	54.4 ± 26.1	70.1 ± 26.3	< 0.001*
Social role functioning	61.1 ± 42.9	68.3 ± 40.1	62.8 ± 42.7	52.4 ± 42.8	72.8 ± 41.2	0.004*
Mental health	57.9 ± 17.2	63.0 ± 17.0	55.9 ± 15.8	55.4 ± 17.1	63.4 ± 17.7	0.001*

*P-value ≤ 0.05

^a^Physical functioning: limitations in physical activities because of health problems;

^b^Physical role functioning: limitations in usual role activities because of physical health problems;

^c^Bodily pain

^d^General Health: general health perception;

^e^Vitality:energy and fatigue;

^f^Social functioning: limitations in social activities because of physical or emotional problems;

^g^Social role functioning: limitations in usual role activities because of emotional problems;

^h^Mental health: general mental health (psychological distress and well-being)

Regarding the self-rated health compared to the previous year, women with concomitant dyspareunia felt that their health has worsened more than other patients (Table 3).

**Table 3:** Self-rated health compared to the previous year (SF-36 question #2)

Answer	All	Superficial dyspareunia	Deep dyspareunia	Superficial and deep dyspareunia	No dyspareunia
	(*n* = 334)	(*n* = 21)	(*n* = 87)	(*n* = 145)	(*n* = 81)
Much better	39 (11.7%)	1 (4.8%)	12 (13.8%)	12 (8.3%)	14 (17.3%)
Somewhat better	51 (15.3%)	5 (23.8%)	11 (12.6%)	24 (16.6%)	11 (13.6%)
About the same	169 (50.6%)	11 (52.4%)	49 (56.3%)	64 (44.1%)	45 (55.6%)
Somewhat worse	59 (17.7%)	4 (19.0%)	14 (16.1%)	32 (22.1%)	9 (11.1%)
Much worse	16 (4.8%)	0 (0.0%)	1 (1.1%)	13 (9.0%)	2 (2.5%)

^a^Note: The Kruskal–Wallis test detected a significant difference between the four study groups (*P*-value = 0.04)

^b^*SF-36* 36-item short form survey

In women suffering from dyspareunia the analysis of Spearman’s rank correlation coefficients (ρ), which correlates symptoms severity and the subscales scores of the SF-36 questionnaire, showed a significant association between low questionnaire scores and high NRS scores, demonstrating that the severity of symptoms correlated with a decline in QoL. As shown in Supplementary Table 2, this association was particularly evident in women with concomitant dyspareunia, which had significant correlations across all domains: physical functioning, physical role functioning, bodily pain, general health, vitality, social functioning, social role functioning, and mental health.

### Sexual function assessment

Total FSFI scores and domain scores were calculated, excluding women who indicated a 0 score on any FSFI item [22] (Table 4). Women with concomitant dyspareunia reported worse sexual function in terms of satisfaction (P =  < 0.001) and pain (P = 0.001). Moreover, arousal scores were lower in patients with dyspareunia (concomitant or isolated) compared to those with no dyspareunia (P = 0.043).

**Table 4:** Mean ± standard deviation of the Female Sexual Function Index (FSFI) questionnaire scorings, overall and by study group

Scale	*n*^*a*^	All	Superficial dyspareunia	Deep dyspareunia	Deep and superficial dyspareunia	No dyspareunia	*P*-value
Desire	249	4.0 ± 1.6	3.5 ± 1.1	4.1 ± 1.5	3.9 ± 1.8	4.1 ± 1.7	0.267
Arousal	292	11.9 ± 5.3	11.5 ± 5.4	11.1 ± 4.2	11.6 ± 5.9	13.3 ± 5.0	0.043*
Lubrication	233	12.0 ± 5.3	11.7 ± 5.3	10.6 ± 4.3	12.0 ± 5.5	13.3 ± 5.5	0.066
Orgasm	246	9.1 ± 3.9	9.1 ± 4.1	8.3 ± 3.3	9.1 ± 4.0	9.8 ± 4.0	0.445
Satisfaction	258	8.0 ± 2.7	8.5 ± 3.1	8.1 ± 2.4	7.2 ± 2.7	9.2 ± 2.5	< 0.001*
Pain	234	10.1 ± 4.0	9.4 ± 4.6	8.2 ± 4.1	10.8 ± 3.3	11.0 ± 5.2	0.001*
Total^b^	77	17.9 ± 5.1	13.6 ± 2.5	17.3 ± 4.8	18.5 ± 5.3	17.9 ± 5.1	0.220

*P-value ≤ 0.05

^a^The analysis of total and relevant domain scores was limited to women who had not indicated a zero score on any of the FSFI items (Meston et al., 2016)

^b^Calculated as Desire × 0.6 + Arousal × 0.3 + Lubrication × 0.3 + Orgasm × 0.4 + Satisfaction × 0.4 + Pain × 0.4 (Meston et al. 2016)

A comparison between sexually active (228/334, 68.3%) and not sexually active women (106/334, 31.7%) revealed that not sexually active women more frequently reported chronic pelvic pain, more severe dyspareunia (NRS median [IQR] 8 [5 to 8] vs 6 [4 to 8], P = 0.008), and worse QoL in all SF-36 domains except physical functioning (Supplementary Table 3–5). Among not sexually active women, 63 (59.4%) cited pain as the reason for not engaging in sexual intercourse, 34 (32.1%) had no partner and did not masturbate, and 9 (8.5%) did not specify a reason.

## Discussion

This study showed a high prevalence of dyspareunia among women with endometriosis, in line with previous literature [7, 13, 23]. Notably, our findings highlight that the majority of these women experience concomitant deep and superficial dyspareunia, which correlates with poorer quality of life (QoL) and sexual function. Furthermore, most women reporting both types of dyspareunia indicated that deep dyspareunia appeared first.

Endometriosis-associated dyspareunia is often described as deep, resulting from contact with sensitive pelvic structures like the pouch of Douglas, cervix, uterus, pelvic floor, and bladder base during deep penetration [24, 25]. Superficial dyspareunia, however, is less frequently investigated and likely underreported in women with endometriosis [10, 23]. Yong et al. reported a 44% prevalence of concomitant dyspareunia in a study of 150 women with provoked vestibulodynia, associating the condition with interstitial cystitis, endometriosis, depression, and higher dyspareunia scores [26]. They hypothesized that these factors contribute to the etiology of concomitant dyspareunia and deep dyspareunia by increasing the risk of nervous system sensitization [26–29]. This hypothesis aligns with our findings, where higher NRS scores for all endometriosis-associated symptoms were noted in patients with concomitant dyspareunia. Specifically, chronic pelvic pain was more prevalent and severe in patients with isolated deep dyspareunia and concomitant dyspareunia, suggesting that chronic pain states characterized by sensitization can lead to regional allodynia and hyperalgesia [30].

An interesting aspect of our findings is that most women with concomitant dyspareunia reported superficial dyspareunia developing after deep dyspareunia. Clinically, this sequence is plausible: women may initially experience deep pain due to a posterior compartment nodule and subsequently develop superficial pain associated with pelvic floor hypertonia as a defensive response [12, 31]. Additionally, local nerve growth (neurogenesis or neuroproliferation) in pelvic structures may sensitize these areas and contribute to both deep and superficial dyspareunia [32].

Our data did not reveal any differences related to the use or type of hormonal therapy, despite reports linking hormonal contraception to secondary vestibulodynia and superficial dyspareunia [33]. The impact of hormonal contraceptives on sexual function is controversial, with mixed evidence regarding their effects on sexual response, desire, lubrication, orgasm, and relationship satisfaction. The mechanisms behind reported sexual difficulties, such as reduced desire and vulvovaginal atrophy, remain unclear, and there is insufficient evidence on their correlation with pelvic floor function [34].

All patients reported a decline in QoL, with the most severe impacts observed in women with concomitant dyspareunia. These women had worse scores across all SF-36 domains: physical function, bodily pain, general health, vitality, mental health, physical role functioning, social role functioning, and social functioning. A significant correlation existed between symptom severity and reduced QoL in all domains for women with concomitant dyspareunia. Superficial dyspareunia notably affected general health, while deep dyspareunia particularly impacted vitality and mental health. This underscores the profound impact of pain on QoL and the importance of assessing pain severity [7].

Additionally, pain severity was associated with reduced sexual activity: non-sexually active women reported more frequently chronic pelvic pain and severe dyspareunia. QoL was also significantly worse for non-sexually active women, highlighting the critical role of sexual health in overall QoL [35].

To our knowledge, this is the first study to use the SF-36 questionnaire to assess the impact of dyspareunia on the QoL of women with endometriosis. However, the association between endometriosis and reduced QoL is well documented [36, 37]. Studies suggest that women treated in tertiary care centers may report worse QoL due to the greater severity of their disease and symptoms [38, 39]. This could explain the reduced QoL reported by all women in our study, possibly reflecting the severity of cases seen at tertiary care centers.

Consistent with existing literature, our data show that women with endometriosis have reduced sexual function [39–43]. Sexual function was particularly impaired in women with concomitant dyspareunia, affecting satisfaction and pain levels more than in those with isolated dyspareunia or no dyspareunia. Tripoli et al. reported that 40% of women with endometriosis were sexually dissatisfied, experiencing lower frequency of sexual intercourse, vaginismus, sexual aversion, and reduced sensuality [40]. Verit et al. found that 69.9% of women with chronic pelvic pain had sexual dysfunction, often due to anxiety about intercourse-related pain, leading to issues like lack of lubrication [44]. A recent study highlighted worsening sexual function associated with severe deep and superficial dyspareunia, independent of pelvic pain, demographic factors, and psychological comorbidities [23]. In our study, arousal scores were lower in patients with dyspareunia (concomitant or isolated) compared to those without dyspareunia. Shum et al. emphasized that both deep and superficial dyspareunia independently worsen sexual QoL, underscoring the importance of treating both conditions [23]. Addressing both superficial and deep dyspareunia in women with endometriosis may be crucial for two reasons. First, different therapeutic strategies may be beneficial: superficial dyspareunia, often associated with pelvic floor hypertonia, can improve with pelvic floor physiotherapy [10, 45], while deep dyspareunia may benefit from surgical treatment to excise endometriotic nodules [46–48], improving sexual satisfaction, desire, and reducing pelvic pain, especially when combined with postoperative medical treatment [49]. Second, understanding the temporal relationship between symptoms may allow early treatment of isolated dyspareunia, particularly deep dyspareunia, potentially preventing the onset of concomitant dyspareunia, which is associated with worse QoL and sexual function.

### Strengths and limitations

The primary strength of this study lies in its novel differentiation between deep and superficial dyspareunia in women with endometriosis. This distinction allows for a more nuanced understanding of the condition and its impact on women’s lives. However, there are some limitations to consider. Firstly, the study was conducted in tertiary care centers, which may mean that the participants had more severe disease and symptoms compared to the general population. Secondly, the study did not specify whether patients with superficial dyspareunia had underlying conditions such as atrophy, vestibulodynia, or pelvic floor hypertonicity, which could play a role in the pathogenesis of the symptom. These areas warrant further research.

## Conclusion

Our data confirm that dyspareunia is common among women with endometriosis. The novelty of our study is that most women reported experiencing both deep and superficial dyspareunia, with deep dyspareunia more frequently occurring first. Women with concomitant dyspareunia exhibited worse quality of life (QoL) and sexual function compared to those with isolated dyspareunia or no dyspareunia. This highlights the need for a comprehensive approach to diagnosis and treatment that addresses both types of dyspareunia to improve the overall well-being of women with endometriosis.

## Supplementary Information

Below is the link to the electronic supplementary material.

Supplementary file1 (DOCX 21 kb)

## Data Availability

Data regarding any of the subjects in the study has not been previously published unless specified. Data will be made available to the editors of the journal for review or query upon request.
