# Supplementary material for: Painful sexual intercourse, quality of life and sexual function in patients with endometriosis: not just deep dyspareunia
Source: Arch Gynecol Obstet. 2024 Jul 25;310(4):2091–100. doi: 10.1007/s00404-024-07643-7 (PMC11392973; doi:10.1007/s00404-024-07643-7)
Supplement: Supplementary file 1 — Supplementary file1 (DOCX 21 kb) [file 404_2024_7643_MOESM1_ESM.docx]

**Supplementary Table 1:**  Severity of dyspareunia-associated symptoms, overall and by study group in women suffering from dyspareunia.

| Characteristic | All | Isolated Superficial dyspareunia | Isolated  Deep dyspareunia | Concomitant Deep and superficial dyspareunia | *P-value* |
| --- | --- | --- | --- | --- | --- |
|  | (*n* = 253) | (*n* = 21) | (*n* = 87) | (*n* = 145) |  |
| NRS, median [IQR] | 7 [4 to 8] | 5 [3 to 6] | 4 [3 to 6] | 8 [6 to 9] | <0.001* |
| NRS class |  |  |  |  | <0.001* |
| 1 to 5 | 92 (36.4%) | 14 (66.7%) | 54 (62.1%) | 24 (16.6%) |  |
| 6 to 10 | 161 (63.6%) | 7 (33.3%) | 33 (37.9%) | 121 (83.4%) |  |

**P*-value ≤0.05.

a) *Note:* Patients with both deep and superficial dyspareunia were assigned the higher of the two scores.

b) *NRS*, numeric rating scale; *IQR*, interquartile range.

**Supplementary Table 2:** Spearman’s rank correlation coefficients (ρ) expressing the association between severity of symptoms (NRS scores) and 36-Item Short Form Survey (SF-36) subscale scorings, overall and by study group in women suffering from dyspareunia.

| Scale | All | Isolated Superficial dyspareunia | Isolated Deep dyspareunia | Concomitant Deep and superficial dyspareunia |
| --- | --- | --- | --- | --- |
|  | (*n* = 253) | (*n* = 21) | (*n* = 87) | (*n* = 145) |
| Physical functioning | –0.335* | –0.048 | –0.251* | –0.307* |
| Physical role functioning | –0.308* | –0.194 | –0.209 | –0.281* |
| Bodily pain | –0.260* | 0.183 | –0.183 | –0.207* |
| General health | –0.235* | 0.266 | –0.218* | –0.270* |
| Vitality | –0.250* | 0.089 | –0.189 | –0.243* |
| Social functioning | –0.251* | –0.187 | –0.144 | –0.171* |
| Social role functioning | –0.234* | –0.175 | –0.193 | –0.186* |
| Mental health | –0.161* | –0.158 | –0.036 | –0.221* |

**P*-value ≤0.05.

*Note:* Patients with both deep and superficial dyspareunia were assigned the higher of the two scores.

*NRS*, numeric rating scale.

1. Physical functioning: limitations in physical activities because of health problems;
2. Physical role functioning: limitations in usual role activities because of physical health problems;
3. Bodily pain;
4. General Health: general health perceptions;
5. Vitality (energy and fatigue);
6. Social functioning: limitations in social activities because of physical or emotional problems;
7. Social role functioning: limitations in usual role activities because of emotional problems;
8. Mental health: general mental health (psychological distress and well-being);

**Supplementary Table 3** Severity of dyspareunia-associated symptoms by sexual intercourses (patients not suffering from dyspareunia are excluded).

| Characteristic | No | Yes | *P*-value |
| --- | --- | --- | --- |
|  | (*n* = 78) | (*n* = 175) |  |
| NRS, median [IQR] | 8 [5 to 8] | 6 [4 to 8] | 0.008* |
| NRS class |  |  | 0.07 |
| 1 to 5 | 22 (28.2%) | 70 (40.0%) |  |
| 6 to 10 | 56 (71.8%) | 105 (60.0%) |  |

**P*-value ≤0.05.

a) *Note:* Patients with both deep and superficial dyspareunia were assigned the higher of the two scores.

b) *NRS*, numeric rating scale; *IQR*, interquartile range.

**Supplementary Table 4.** Characteristic of the study sample by sexually active women

| Characteristic | No | Yes | *P*-value |
| --- | --- | --- | --- |
|  | (*n* = 106) | (*n* = 228) |  |
| Age group, y |  |  | 0.46 |
| ≤35 | 29 (27.4%) | 64 (28.1%) |  |
| 36 to 40 | 26 (24.5%) | 62 (27.2%) |  |
| 41 to 45 | 21 (19.8%) | 55 (24.1%) |  |
| >45 | 30 (28.3%) | 47 (20.6%) |  |
| BMI class, kg/m² |  |  | 0.90 |
| <25 | 79 (74.5%) | 168 (73.7%) |  |
| 25 to <30 | 19 (17.9%) | 39 (17.1%) |  |
| ≥30 | 8 (7.5%) | 21 (9.2%) |  |
| Type of endometriosis |  |  |  |
| Adenomyosis | 61 (57.5%) | 119 (52.2%) | 0.41 |
| Posterior DIE | 41 (38.7%) | 93 (40.8%) | 0.81 |
| Ovarian | 33 (31.1%) | 73 (32.0%) | 0.90 |
| Anterior DIE | 0 (0.0%) | 6 (2.6%) | 0.18 |
| Other | 4 (3.8%) | 6 (2.6%) | 0.73 |
| DIE site |  |  |  |
| Rectum | 27 (25.5%) | 66 (28.9%) | 0.60 |
| Uterosacral ligaments | 15 (14.2%) | 30 (13.2%) | 0.86 |
| Sigma | 11 (10.4%) | 21 (9.2%) | 0.84 |
| Torus | 6 (5.7%) | 9 (3.9%) | 0.57 |
| Rectovaginal septum | 2 (1.9%) | 6 (2.6%) | 1.00 |
| Vagina | 0 (0.0%) | 6 (2.6%) | 0.18 |
| Bladder | 0 (0.0%) | 5 (2.2%) | 0.18 |
| Ureters | 0 (0.0%) | 2 (0.9%) | 1.00 |
| Parametrium | 1 (0.9%) | 5 (2.2%) | 0.67 |
| HT |  |  | 0.62 |
| No | 34 (32.1%) | 80 (35.1%) |  |
| Yes | 72 (67.9%) | 148 (64.9%) |  |
| Type of HT |  |  | 0.40 |
| No HRT | 34 (32.1%) | 80 (35.1%) |  |
| E/P | 20 (18.9%) | 41 (18.0%) |  |
| P | 44 (41.5%) | 78 (34.2%) |  |
| LNG-IUD | 8 (7.5%) | 29 (12.7%) |  |
| Previous surgery |  |  | 0.55 |
| No | 41 (38.7%) | 97 (42.5%) |  |
| Yes | 65 (61.3%) | 131 (57.5%) |  |
| Type of surgery |  |  | 0.08 |
| No surgery | 41 (38.7%) | 97 (42.5%) |  |
| Ovarian | 29 (27.4%) | 45 (19.7%) |  |
| DIE | 8 (7.5%) | 37 (16.2%) |  |
| Ovarian and DIE | 28 (26.4%) | 48 (21.1%) |  |
| Superficial | 0 (0.0%) | 1 (0.4%) |  |
| Previous pregnancies |  |  | 0.004* |
| No | 98 (92.5%) | 182 (79.8%) |  |
| Yes | 8 (7.5%) | 46 (20.2%) |  |
| Smoker |  |  | 0.51 |
| No | 80 (75.5%) | 163 (71.5%) |  |
| Yes | 26 (24.5%) | 65 (28.5%) |  |
| Dysmenorrhea, NRS |  |  | 0.93 |
| 0 | 56 (52.8%) | 116 (50.9%) |  |
| 1 to 5 | 17 (16.0%) | 41 (18.0%) |  |
| 6 to 10 | 33 (31.1%) | 71 (31.1%) |  |
| Dyschezia, NRS |  |  | 0.08 |
| 0 | 72 (67.9%) | 173 (75.9%) |  |
| 1 to 5 | 12 (11.3%) | 29 (12.7%) |  |
| 6 to 10 | 22 (20.8%) | 26 (11.4%) |  |
| Dysuria, NRS |  |  | 0.58 |
| 0 | 91 (85.8%) | 201 (88.2%) |  |
| 1 to 5 | 7 (6.6%) | 16 (7.0%) |  |
| 6 to 10 | 8 (7.5%) | 11 (4.8%) |  |
| Chronic pelvic pain, NRS |  |  | 0.03* |
| 0 | 51 (48.1%) | 145 (63.6%) |  |
| 1 to 5 | 27 (25.5%) | 39 (17.1%) |  |
| 6 to 10 | 28 (26.4%) | 44 (19.3%) |  |
| Ovulation pain, NRS |  |  | 0.37 |
| 0 | 66 (62.3%) | 151 (66.2%) |  |
| 1 to 5 | 16 (15.1%) | 40 (17.5%) |  |
| 6 to 10 | 24 (22.6%) | 37 (16.2%) |  |

**P*-value ≤0.05.

a) *BMI*, body mass index; *HT*, hormone therapy; *E/P*, combined estrogen-progesterone; *P*, progesterone; *LNG-IUD*, levonorgestrel-releasing intrauterine device; *DIE*, deep infiltrating endometriosis; *NRS*, numerical rating scale.

**Supplementary Table 5.** Mean ± standard deviation of eight 36-Item Short Form Survey (SF-36) subscale scorings by sexual intercourses.

| Scale | No | Yes | *P*-value |
| --- | --- | --- | --- |
|  | (*n* = 106) | (*n* = 228) |  |
| Physical functioning | 84.7 ± 21.5 | 87.9 ± 19.9 | 0.19 |
| Physical role functioning | 60.8 ± 40.8 | 71.7 ± 36.4 | 0.02* |
| Bodily pain | 56.9 ± 28.7 | 66.8 ± 26.7 | 0.002* |
| General health | 51.5 ± 20.6 | 56.5 ± 21.4 | 0.04* |
| Vitality | 44.7 ± 17.8 | 50.9 ± 18.0 | 0.003* |
| Social functioning | 55.8 ± 27.5 | 63.6 ± 25.1 | 0.02* |
| Social role functioning | 50.9 ± 44.7 | 65.8 ± 41.3 | 0.003* |
| Mental health | 54.6 ± 17.9 | 59.5 ± 16.7 | 0.02* |

**P*-value ≤0.05.
